# Genetic dissection of plant architecture reveals haplotypes controlling sink-related traits in oilseed rape under limited nitrogen fertilization

**DOI:** 10.1186/s12870-025-07035-2

**Published:** 2025-08-27

**Authors:** Sven Weber, Andreas Eckert, Rod J. Snowdon, Andreas Stahl

**Affiliations:** 1 Department of Plant Breeding, IFZ Research Centre for Biosystems, Land Use and Nutrition, Justus Liebig University, Giessen, Germany; 2 Julius Kühn-Institute (JKI)– Federal Research Center of Cultivated Crops, Institute for Resistance Research and Stress Tolerance, Erwin-Baur-Str. 27, 06484 Quedlinburg, Germany

**Keywords:** Brassica napus, F1 hybrids, Genome wide association study, Primary yield components, Source-sink relationship

## Abstract

**Background:**

Plant architecture and primary yield components strongly influence the sink strength for nitrogen in winter oilseed rape (*Brassica napus*). Their optimization can contribute substantially to enhance nitrogen utilization efficiency, reduce the nitrogen balance surplus and thus reduce negative side effects of oilseed rape cultivation. However, the genetic architecture of individual yield components is not sufficiently understood, and enhanced knowledge could accelerate breeding of more efficient varieties. Here, we manually assessed yield components and plant architectural traits in 323 experimental F1 hybrids derived from crosses between 162 father lines from a winter oilseed rape nested association mapping population and two different maternal testers.

**Results:**

We observed significant genetic effects and differences between the two mothers for all traits. Although the mean number of siliques on side branches showed comparatively little variation, the F1 hybrids from the two different maternal testers differed greatly in their distribution of siliques on the side branches. On the sixth-lowest side branches from the base of the stem (level 6) the number of siliques was correlated with grain yield (*r* >0.3) and showed the highest heritability (h^2^ = 0.289), while heritability for grain yield was h^2^ = 0.414.

**Conclusion:**

By dissecting the genetic architecture of relevant traits, we identified haplotype blocks associated with the regulation of silique number on individual side branches, explaining up to 8% of total phenotypic variation. For certain alleles of block b000301 on chromosome A10, we observed a strong influence on the number of siliques, which ranged from 10.5 to 43.69 for siliques on side branch levels 11–15 and from 56.36 to 107.24 for siliques on side branch levels 6–10. Important haplotype blocks affecting many subtraits simultaneously were found to overlap with QTL found in other studies, emphasizing their relevance for breeding.

**Supplementary Information:**

The online version contains supplementary material available at 10.1186/s12870-025-07035-2.

## Introduction

Nitrogen (N) is the most important plant nutrient for crop fertilization in terms of quantity and it contributes significantly to producing adequate amounts of agricultural commodities which are necessary to meet the demands of a growing world population. Industrial fertilizer production based on the Haber-Bosch process is attributed with feeding up to 50% of the world’s population [1]. However, excess N can escape from the production system into atmospheric, aquatic, marine and terrestrial ecosystems, leading to contamination with nitrate or gaseous volatile losses [2, 3]. The latter contribute significantly to climate change [4]. According to Richardson et al. (2023) [5], the problems associated with massive perturbations of the global nitrogen cycle have already crossed the global boundaries and urgent actions are required to reduce the negative effects of reactive nitrogen [6, 7]. Besides those ecological drawbacks, high fertilizer costs diminish the farmers financial earnings. Hence, enhanced nitrogen use efficiency of agricultural production is one major agricultural megatrend. On the plant level nitrogen use efficiency (NUE) can not only be considered as an overall parameter, rather it can also be decomposed into the contributing components N uptake efficiency (NupE) and N utilization efficiency (NutE) [8].

Winter oilseed rape (OSR) is a comparatively efficient crop in the acquisition of nitrogen, with uptake before winter reaching up to 100 kg N/ha [9]. By the time the maximum biomass is reached at flowering, more than 200 kg N/ha can be accumulated in the plant canopy, with some variation [10]. However, nitrogen which is already taken up in the plant is not efficiently translocated to the seeds, the plant organ which is subsequently harvested, and this results in a high N balance surplus [11, 12]. One reason for the comparatively low translocation of nitrogen from the biomass to the grains in OSR is the asynchronization of the source-sink relationships. During the period when the vegetative biomass peaks during flowering and the large nitrogen-rich leaves senesce, pods into which the nitrogen can be mobilized are not yet developed. The lower leaves are then shed with incomplete remobilization. Later, when the sink (i.e. the growing siliques) is properly formed and mainly contributes to the plant’s photosynthetic performance [13], substantial portions of the source are no longer available due to senescence and dropped leaves. This low nitrogen utilization efficiency (NutE) results in incomplete mobilization from the shed leaves during and shortly after flowering. The high N concentrations in dropped leaves [14] and by the rapid decomposition of shed leaves on the soil surface prior to harvest [15] are indicators of a low nitrogen remobilization. Hence, besides the relevance of root traits [16] and intermediate buffers for N in the stem [17], the sink strength is a key parameter to enhance NutE and NUE [12]. A larger sink is not only the prerequisite for a higher yield, but also enables a larger amount of nitrogen to be incorporated into the seeds, which is then harvested from the field and is therefore not subject to losses.

Several studies provide empirical evidence of breeding progress for higher NUE [18–22]. In this context, it was shown that F1 hybrids have an advantage over pure bred lines [20, 21, 23–25]. By breaking down the yield into the primary yield components, the performance superiority of high yielding and N-efficient varieties was attributed to a higher number of siliques [10]. Simulation studies demonstrated that the most obvious alteration of yield can be achieved by the number of seeds per plant, the thousand seed weight (TSW) and– to a lesser extent– the number of seeds per silique [26]. OSR is known for a very high compensatory ability, which means that deficits in the first developed traits can be subsequently compensated by other characters. For example, a shading experiments demonstrated that the low number of siliques developed can be compensated by a higher TSW at later times (after removal of the shading) [27]. It can be argued that genotypes that have more and/or stronger siliques transfer more of the nitrogen incorporated in the vegetative biomass to the sink, i.e. the harvestable matter, and the nitrogen is then removed from the field instead of being lost.

While the genetic variation presented in the gene pool of *B. napus* was subject of several studies [28–32]primary yield components of a large number of genotypes were rarely described in detail as in the studies mentioned before. Hence, comprehensive field studies on both the composition of the primary yield components under low N as well as the genetic determinants of the respective primary yield components in (winter-type) OSR are limited.

Consequently, the objectives of this study were (i) to assess the genetic variation for primary yield components at very high resolution in a panel of 323 experimental F1 hybrids representing the broad genetic variation in winter OSR, (ii) to investigate their relevance to yield formation, and (iii) to determine the genetic architecture of individual traits. Those information should contribute to a more targeted introgression and stacking of beneficial alleles into elite breeding programs.

## Materials and methods

### Genetic material

For this study 323 experimental F1 hybrids were generated by controlled pollination of two modern, elite, male-sterile maternal parents by paternal doubled haploid (DH) lines or recombinant inbred lines (RIL). All paternal lines were half-sibs from a large *B. napus* nested association mapping (BnNAM) population, which represents the breadth of genetic diversity found within European winter-type OSR [33, 34]. Yield data from a previous study of test crosses with the elite mother MSL007 [34] were used to select the father lines. Only those BnNAM lines that achieved a minimum yield (48.9 dt/ha for the DH lines and 46.2 dt/ha for the RILs) were used as father lines for the new test crosses conducted here. This preselection was intended to exclude genotypes that inherit low yield performance and to avoid trivial findings attributable to long term adaptation and selection among the parental lines. Thereafter, the remaining genotypes were screened for genetic diversity based on Rodgers Distance. If lines were very similar, only one of them was used to generate the test cross hybrids. Based on this strategy, a subset of 162 individuals belonging to 36 NAM families, each comprising 1 to 11 family members, were selected for test cross production. The development of F1 hybrids followed the principle described [34]. However, here each father line was crossed with two distinct mother lines, so that– except for a few failures– two complete experimental F1 hybrids hybrid sets were available for each tester, hereafter referred to as Mother A and Mother B.

### Plant cultivation

All experimental F1 hybrids were cultivated at Justus Liebig University’s Rauischholzhausen field station (50.78, 8.88) in two subsequent years (2019–2020 and 2020–2021 growing seasons) together with 27 modern elite winter OSR varieties that were registered between 2012 and 2020. Each genotype was grown unreplicated in one field plot (1.25 m x 8.4 m). Randomly, 50 genotypes were replicated, so the experimental design can be considered as an incompletely replicated p-rep design [35] with complete randomization within both years. Since it is known that plant structure and primary yield components are strongly dependent on nutrient availability [36], all traits in the present study are investigated in field trials with limited nitrogen fertilization. For N fertilization, the respective Nmin value was included, so that in both years fertilization was applied to an approximately uniform restricted level of 125 kg N/ha. The first N application was fertilized statically with 65 kg N/ha, while the remaining 60 kg N/ha were applied with the second N application minus the specific Nmin value. Application of further nutrients and plant protection measures was carried out as needed to contain side effects caused by pests (insect pests) and diseases (*Sclerotinia sclerotiorum*, *Phoma lingam*) and to realize a high proximity to realistic cultivation conditions.

### Assessment of plant architecture traits

Plant architecture is highly dependent on canopy density and a negative correlation between number of siliques and canopy density is usually expected. Moreover, as differences in winter hardiness might manifest on plant architecture parameters, the post-winter plant density was corrected to a uniform value of 25 plants per m² at a marked position within each plot. By doing so, it was ensured that all plants had the same initial growing conditions during the spring vegetation.

Before grain maturity, five consecutive plants of each genotype were taken from the center of the plot. These plants were manually examined for their plant architectural parameters, including the height of the respective primary branches, the angles of the secondary branches, the number of secondary branches, the number of siliques on the main raceme and the number of siliques per secondary branch. The thousand seed weight (TSW) was determined from the harvested crop of the entire plot.

### Statistical analyses

The phenotypic data were collected manually. A thorough check of the data was carried out. Obviously implausible values were excluded from the analysis. Prior to any statistical analyses the arithmetic mean was calculated for measurements of all architectural traits on a plot level, so that each plot has a single entrance for each architectural trait. Subsequently, the formation of adjusted genotype means, which take into account the design factors of the trial in the model, should provide the best possible data for the downstream analysis. Adjusted genotype means were calculated across the two years with the model described in Eq. 1 .

1 $$\:{y}_{ijkl}=\mu\:+{G}_{i}+{E}_{j}+{G}_{i}{E}_{j}+{R}_{jk}+{C}_{jl}+{e}_{ijkl}$$

Where $\:{y}_{ijkl}$, is the observation of the *i*th genotype in the *j*th year, in the *k*th row and the lth column. $\:\mu\:$ is the fixed intercept and $\:G$ the genotype mean defined as fixed effects. $\:E$ and $\:GE$ are random effects of year and genotype year interaction, $\:R$ and $\:C$ are the random effect of row and column nested in years, lastly $\:e$ is the random residual term. Residuals effects were assumed to be random with.

In addition, adjusted genotype means for each trait were calculated separately for the first and second year of the trial. The same model as described in Eq. 1 was used for this– with the only deviation being that the year effect ($\:E$) was not taken into consideration.

As mentioned above, only 50 genotypes were repeatedly tested in both years, resulting in an unbalanced experimental design. Furthermore, there are plant-architectural traits that are not available for all plants because they did not exist on all individuals due to their phenology (e.g. number of pods on high side branches are not available in case the plant only had a low number of side branches). This further leads to an unbalanced data structure. For these reasons heritabilities were calculated using the following formula, proposed by [37] based on [38]:

2 $$\:{H}^{2}=\frac{{\sigma\:}_{G}^{2}}{{\sigma\:}_{G}^{2}+\raisebox{1ex}{${\stackrel{-}{v}}_{\varDelta\:..}^{BLUE}$}\!\left/\:\!\raisebox{-1ex}{$2$}\right.}$$

Here, $\:{\sigma\:}_{G\:}^{2}$is the genetic variance obtained from a full random model. This model was equivalent to the model described above with the difference that the genotype effects were considered random. While $\:{\stackrel{-}{v}}_{\varDelta\:..}^{BLUE}\:$is the mean variance of pairwise genotype contrasts, which were obtained from the model described above with genotype effects as fixed factors. All computations related to the mixed models to obtain adjusted means and variance components were conducted within the R software [39] using the package “lme4” [40], while $\:{\stackrel{-}{v}}_{\varDelta\:..}^{BLUE}\:$was obtained using a custom R script.

### Genotypic data

All parental lines were genotyped with the Brassica 60k SNP array [41] (Supplementary Table S1). Since many markers on that array do not act in a locus specific manner [42, 43] we applied a stringent filtering based on a BLAST search tool (https://www.ncbi.nlm.nih.gov/books/NBK21097/) to the reference “EXPRESS617” [44] and excluded markers that have multiple positions in the reference genome or exhibit any mismatches in their 50 bp probe sequence. Furthermore, markers with a minor allele frequency ≤ 0.05 and a missing rate of ≥ 5% were discarded. Since DH lines are expected to be completely homozygous, we considered any heterozygous marker call also as missing call. This left us with a total of 12,001 high quality markers for further analysis.

### Genome-wide association study

To identify SNP markers that exert a significant influence on architectural traits, a comprehensive Genome-Wide Association Study (GWAS) was conducted, focusing on architectural traits with a H^2^ ≥ 0.1. The mixed linear model proposed by [45] was utilized for GWAS, testing each marker sequentially.

3 $$\:y=X\beta\:+Mu+Zg+e$$

here, $\:y$ represents the vector of adjusted means for a given architectural trait. $\:X$ is the design matrix for fixed effects, which includes the overall mean, a fixed effect accounting for the hybrid mothers, and the incorporation of the first three principal components of the marker data to address population stratification, along with their effects $\:\beta\:$. $\:M\:$corresponds to the design matrix for the marker under examination along with its fixed effect $\:u$. $\:Z\:$is the design matrix of the random genotype effects $\:g$, while $\:e$ is the random residual term.

It is assumed that $\:g\sim\:N\left(0{,G\sigma\:}_{g}^{2}\right)$. and $\:e\sim\:N(0,I{\sigma\:}_{e}^{2})$.

The genomic relationship matrix, following [46], is calculated as.

4 $${\boldsymbol G}_{\mathbf a}\boldsymbol=\frac{\mathbf M\mathbf M\boldsymbol'}{2\sum\rho_i\left(1-\rho_i\right)}$$

The elements of ***M*** are represented by (0-2p_i_) for homozygous allele A, (1-2p_i_) for the heterozygous state, and (2-2p_i_) for homozygous allele B, $\:{p}_{i}$ with being the allele frequency of the B allele. The r package sommer [47] was employed for GWAS. A significance threshold of p-value ≤ 10 − 3 (-log(p-value) ≥ 3) was chosen for identifying marker–trait associations. This cutoff is widely employed in plant breeding research ([51]. Furthermore, adopting p-value ≤ 10 − 3 also enabled us to explore potential overlaps of QTL regions identified in our study with those reported in other investigations on rapeseed architecture [48–52] in which the threshold was set p-value ≤ 10 − 3.

### Haplotype blocks

Linkage disequilibrium (LD) between multiple markers and QTL results in multiple GWAS hits around the underlying QTL. To better pinpoint the genomic region with significant influence on the trait under examination, haplotype blocks were constructed. Here, LD between all markers was calculated as *r*^*2*^ in the R package “SelectionTools” (available at http://population-genetics.uni-giessen.de/~software/*).* Subsequently, haplotype blocks were built by starting with the two neighboring markers with the highest LD. If the pairwise LD exceeded a threshold of *r*^*2*^ ≥ 0.4, those markers were then assigned to a haplotype block. In the next step, if the LD between the next immediately adjacent markers and the markers at the block border again exceeded the threshold, the block was extended. Again, since many markers on that array did not act in a locus specific manner [42, 43] which was also observable in the LD pattern, resulting in a checkboard-like pattern in the LD plot (Supplementary Fig. 1). To accommodate this, a tolerance parameter of 12 markers was integrated into the haplotype block construction process. This parameter allowed for the acceptance of 12 markers that did not meet the LD threshold if the 13th flanking marker fulfilled the LD criterion again. If one of the markers inside of the block was significant in the GWAS, the whole block was deemed significant.

### Variance explained by haplotype block

The proportion of phenotypic variance explained by a haplotype block with a significant trait association was calculated using the following mixed linear model:

5 $$y=X\beta+Z_bg_b+Z_rg_r+e$$

here,$\:\:y$ represents the vector of adjusted means for a given architectural trait. While $\:X\:$is the design matrix for the fixed effects $\:\beta\:$ and contains all fixed effects described in the GWAS section except the effect of the significant marker, $\:{Z}_{b}\:$is the design matrix for the random effects of markers within a significant haplotype block with their associated effects $\:{g}_{b},$ while $\:{Z}_{r}$ is the design matrix for all remaining markers with their random effect $\:{g}_{r}$. It is assumed that $\:{g}_{b}\sim\:N\left(0{,{G}_{b}\sigma\:}_{gb}^{2}\right)$, $\:{g}_{r}\sim\:N\left(0{,{G}_{r}\sigma\:}_{gr}^{2}\right)$ and $\:e\sim\:N\left(0{,I\sigma\:}_{e}^{2}\right)$. The genomic relationship matrices $\:{G}_{b}\:$and $\:{G}_{r}\:$ were calculated following the method by van Raden as described above using markers within a significant haplotype block and all remaining markers respectively. The variance explained by a significant haplotype block is then calculated as: 

6 $$\:\frac{{\sigma\:}_{gb}^{2}}{{\sigma\:}_{gr}^{2}+{\sigma\:}_{e}^{2}}$$

This procedure was performed for each significant haplotype block, respectively.

### Overlap with plant architecture relevant QTL in literature

Several studies have sought to unravel the genetic regulation of plant architecture in rapeseed [52–57]. In order to assess the overlap of QTL identified in existing literature with trait-associated haplotype blocks discovered in our current study, we examined the placement of the reported QTL on the *B. napus* EXPRESS617 reference genome reference genome [40]. For studies utilizing the Brassica 60k SNP array, we determined the position of the reported significant marker with the lowest e-value on the EXPRESS617 reference genome via BLAST analysis, as detailed above. In cases where alternative genotyping tools were employed, we obtained the marker sequences of significantly associated markers from the respective studies and performed a BLAST analysis against the EXPRESS617 reference [44]. Subsequently, we utilized the hit with the lowest e-value as the QTL position on EXPRESS617. A QTL was deemed to overlap with those identified in our study if its position fell within the boundaries of a significant haplotype block.

## Results

### Genetic variation for seed yield

To evaluate the potential of a broad genetic variation for improving the sink capacity of F1 Hybrids in OSR, a total of 323 experimental F1 hybrids together with 27 elite hybrid cultivars were examined for yield performance, primary yield components and plant architectural characteristics. 162 experimental F1 hybrids originated from mother A and 161 from mother B. The average yield across all genotypes tested was 36.76 t/ha with a coefficient of variation of 8.2%. Examination of the adjusted yield means (on the plot level) revealed that experimental F1 hybrids tracing back to mother A had lower yield performance than those tracing back to mother B (3.534 vs. 3.726 t/ha).

### Primary yield components

The mean number of siliques per plant was 128.69 but showed a considerable variation of 26.7% between the genotypes. In contrast, the average TSW of 4,335 g with a coefficient of variation of 3.8% only showed a small variation between the tested genotypes (Table 1). 

The average number of siliques per side branch varied between 11.73 on the lowest side shoot and 22.27 on side branch level 7, with side branch level 1 showing the lowest broad-sense heritability (h^2^ = 3.1%) and side branch level 5 the highest at h^2^ = 28.9%. The number of siliques on the side branches of experimental F1 hybrids from mother B was always 1.76 (for side branch 1) to 5.74 (for side branch 4) higher than for hybrids from mother A. In contrast, across all experimental F1 hybrids, the main branch contained an average of 3.25 siliques more on hybrids going back to mother A than on hybrids from mother B (Table 1).

**Table 1:** Heritability, population mean, coefficient of variation (calculated as standard deviation/population mean), mean separated according to the test mother A vs. B and significance level of t-test between both tester for seed yield, primary yield components and all architectural traits

Trait		H^2^	Population mean	Coefficient of variation	Mean of mother A	Mean of mother B	p-value t-test between mothers
Seed yield		0.414	3.676	0.082	3.534	3.726	<0.001***
Primary yield components	Siliques plant^-1^	0.076	128.69	0.267	128.863	128.338	0.909
	TSW [g]	0.297	4.335	0.038	4.274	4.381	<0.001***
Height	Side branch 1 [cm]	0.13	36.425	0.193	36.87	35.139	0.039*
	Side branch 2 [cm]	0.105	43.146	0.161	43.604	41.881	0.039*
	Side branch 3 [cm]	0.144	48.969	0.146	49.395	47.627	0.037*
	Total [cm]	0.178	123.103	0.051	120.744	124.595	<0.001***
Angle	Side branch 1	0	20.902	0.144	21.243	20.354	0.018*
	Side branch 2	0	21.323	0.167	21.641	20.734	0.039*
	Side branch 3	0	21.989	0.171	22.127	21.57	0.254
Siliques	Side branch 1	0.031	11.728	0.368	10.816	12.576	<0.001***
	Side branch 2	0.071	14.656	0.319	12.709	16.589	<0.001***
	Side branch 3	0.122	18.12	0.276	15.997	20.139	<0.001***
	Side branch 4	0.216	20.437	0.274	17.387	23.125	<0.001***
	Side branch 5	0.289	21.354	0.254	18.38	23.763	<0.001***
	Side branch 6	0.15	22.238	0.222	19.675	24.209	<0.001***
	Side branch 7	0.12	22.263	0.248	19.859	23.986	<0.001***
	Side branch 8	0.221	22.267	0.238	19.979	23.926	<0.001***
	Side branch 9	0.284	22.228	0.257	20.301	23.761	<0.001***
	Side branch 10	0.205	21.901	0.297	20.364	22.908	<0.001***
	Side branch 11	0.013	21.84	0.356	19.95	23.202	<0.001***
	Side branch 12	0	21.618	0.354	20.18	22.913	0.004**
	Side branch 13	0.083	21.325	0.35	20.357	21.756	0.2
	Side branch 14	0.124	20.737	0.373	20.132	21.236	0.427
	Side branch 15	0	20.801	0.368	19.809	22.801	0.07
	Main branch	0.227	39.819	0.145	41.486	38.233	<0.001***
	Siliques on side branches plant^-1^	0.076	128.395	0.267	128.65	127.977	0.909
	Siliques plant^-1^	0.076	128.69	0.267			0.909
	Side branch 1 to 5	0.21	82.933	0.23	72.385	92.388	<0.001***
	Side branch 6 to 10	0.127	70.579	0.318	65.369	74.778	<0.001***
	Side branch 11 to 15	0.121	16.201	0.806	19.415	13.761	<0.001***
	Side branch 16 to 20	0.065	1.313	2.395	2.144	0.612	0.463
Secondary branches	Side branch 1	0.088	0.334	1.231	0.335	0.323	0.863
	Side branch 2	0	0.368	1.049	0.345	0.391	0.38
	Side branch 3	0.012	0.479	0.912	0.455	0.485	0.616
	Side branch 4	0	0.535	0.799	0.441	0.62	<0.001***
	Side branch 5	0.116	0.552	0.818	0.473	0.608	0.013*
	Side branch 6	0	0.496	0.773	0.445	0.519	0.123
	Side branch 7	0.067	0.452	0.936	0.399	0.476	0.129
	Side branch 8	0	0.414	1.043	0.347	0.461	0.036*
	Side branch 9	0.077	0.355	1.113	0.308	0.393	0.078
	Side branch 10	0.273	0.308	1.456	0.275	0.321	0.45
	Side branch 11	0	0.248	1.634	0.186	0.291	0.037*
	Side branch 12	0.053	0.225	2.007	0.145	0.27	0.029*
	Side branch 13	0	0.204	1.99	0.169	0.198	0.647
	Side branch 1 to 5	0	1.246	0.67	0.995	1.453	<0.001***
	Side branch 6 to 10	0.017	0.903	0.814	0.836	0.895	0.521
	Side branch 11 to 15	0	0.132	1.543	0.118	0.139	0.427
	Side branch 15 to 20	0	0.007	5.677	0.011	0.002	0.078

### Heritability of investigated traits

Based on the heritability of the traits, it is measured how strongly a trait has been influenced by genetics relative to the external environmental factors. Heritability of the of the architectural traits ranged from h^2^ = 0 for side branch angles to h^2^ = 28.9% for the number of siliques on the 5th side branch from the stem base. Among the primary yield components, TSW had the highest broad sense heritability with 29.7% on average (Table 1). It is to note that in contrast to the number of siliques on the side branches, it was found that the number of secondary branches on the side branches was predominantly non-heritable and the values fluctuated mainly around 0, with only side branches levels 5 and 11 showing a certain degree of heritability at h^2^ = 11.6 and h^2^ = 27.3% respectively. Therefore, the number of secondary branches were not included in further genetic analysis.

### Inter-trait correlations

**Fig. 1:**
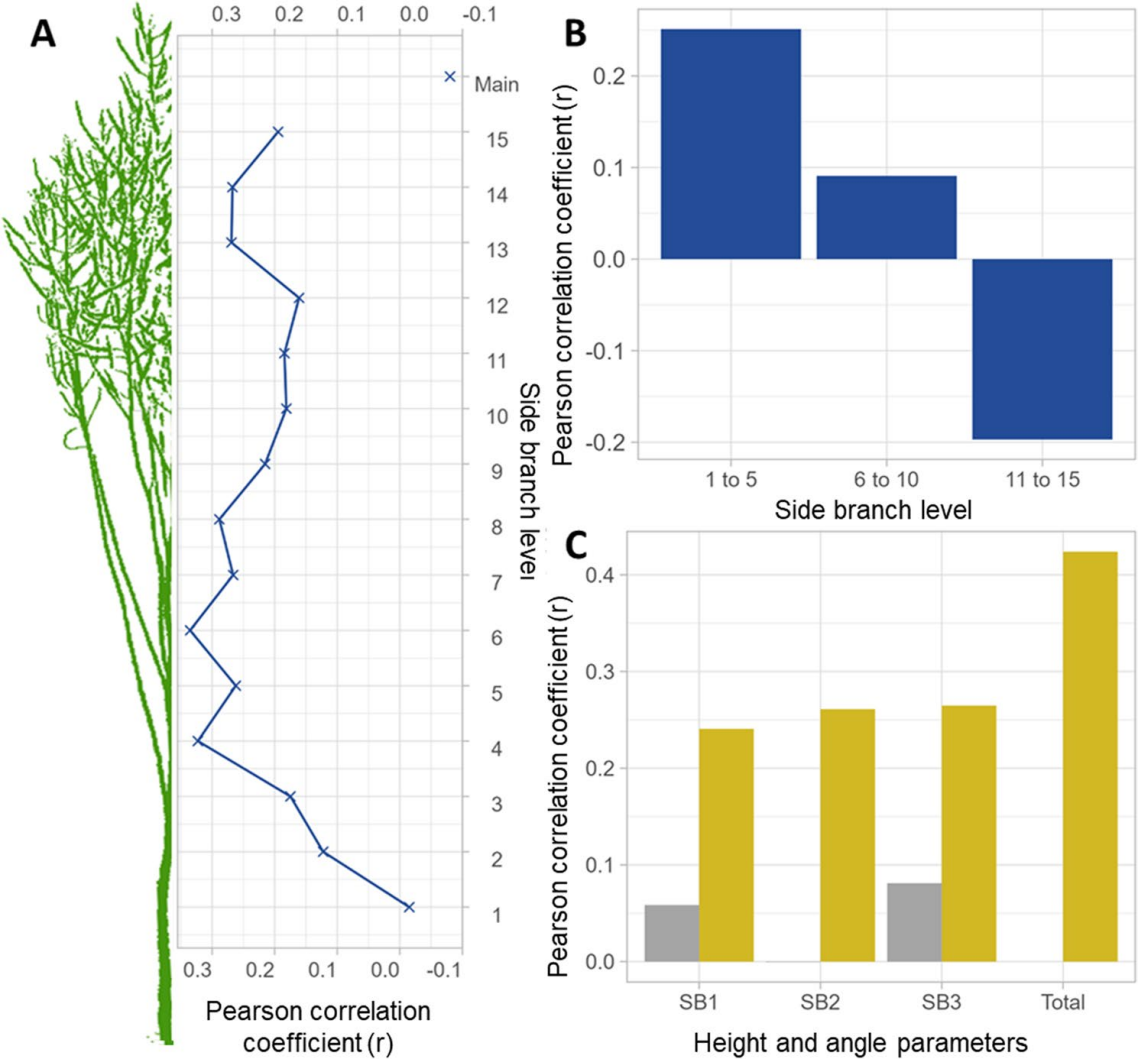
Pearson coefficient of correlation (r) of the plant architecture traits to seed yield on the plot level. **A** Individual correlation of number of siliques to seed yield according to the branch level along the vertical plant axis showing that the number of siliques on the mid-level side branches (6–10) have the strongest correlation to seed yield on the plot level. **B** Correlation of aggregated number of siliques on five subsequent side branches to seed yield on the plot level. **C** Correlation of height (yellow) and angle (grey) to seed yield on the plot level according to the individual level from low to higher side branches (SB)

**Fig. 2:**
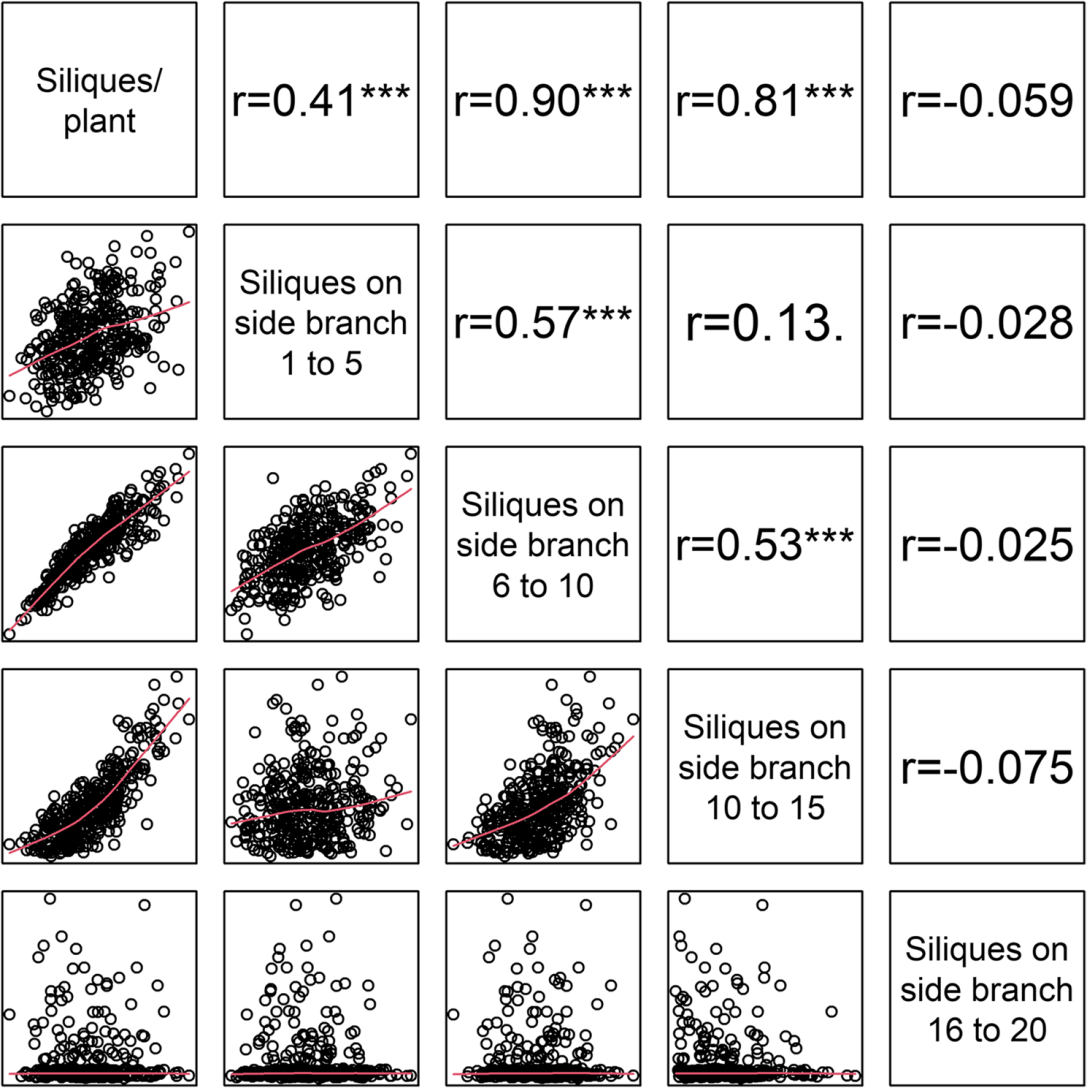
Pearson correlation of correlation among number of siliques in different zones of the siliques package and with total number of siliques per plant

By using the Pearson coefficient of correlation to examine the characteristic relationship between the number of siliques on side branches and the grain yield, it can be seen that correlations increase from the lower end of the siliques bundle up to the 4^th^ level, then remain at a higher level from the 4^th^ to 6^th^ level but decrease from the 7^th^ level upwards. For the number of siliques on the main raceme no significant. correlation could be determined with grain yield, (Fig. 1). Although the characteristic expression for each side branch was recorded separately and the data are therefore independent, it can be argued that the characteristics on the directly adjacent or next higher/lower side branches are not completely unrelated and were affected by environmental factors in a similar manner during plant development. Therefore, further analysis was carried out in a combined form of five consecutive side branch levels. It was shown that siliques at all zones (1–5, 6–10, 10–15 and 16–20) correlated with the total number of siliques per plant, with the middle levels showing more than twice the correlation (*r* = 0.9 and 0.81) than the lower and above siliques level 5 (Fig. 2).

### Genome wide association studies

####  Genome wide association studies for adjusted means across both years

Leveraging all 12,001 high-quality SNP markers, we successfully identified 49 haplotype blocks associated with traits related to silique numbers (Supplementary Fig. 2, Supplementary Tables S2 and S3). These blocks were distributed across all rapeseed chromosomes, except for chromosome A01 and A04. Most of the identified haplotype blocks exhibited significant association for a single trait (Supplementary Table S3). It has to be mentioned that, in general, these blocks explained a modest portion of the phenotypic variation, with the highest percentage explained by block b000563 on chromosome C06 for siliques on side branch 13 (9.16%, Supplementary Table S2).

**Fig. 3:**
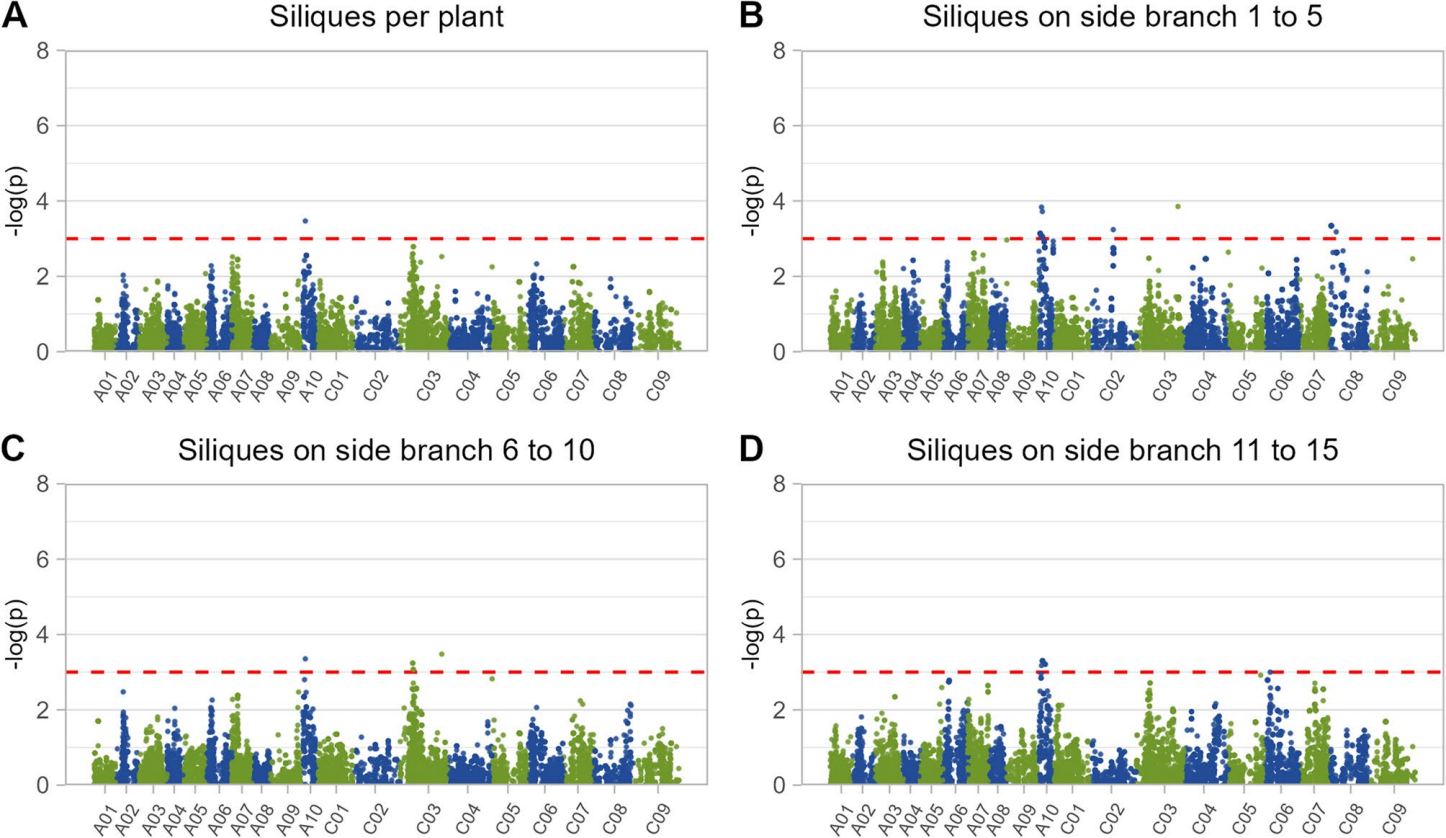
Manhattan plot of genome wide association study (GWAS) based on 12,001 genome wide single nucleotide marker for (**A**) number of siliques per plant, (**B**) Siliques on side branches 1–5, (**C**) siliques on side branches 6–10, and (**D**) siliques on side branches 11–15. Red line represents arbitrary significant threshold of *p*-value = 0.001

**Fig. 4:**
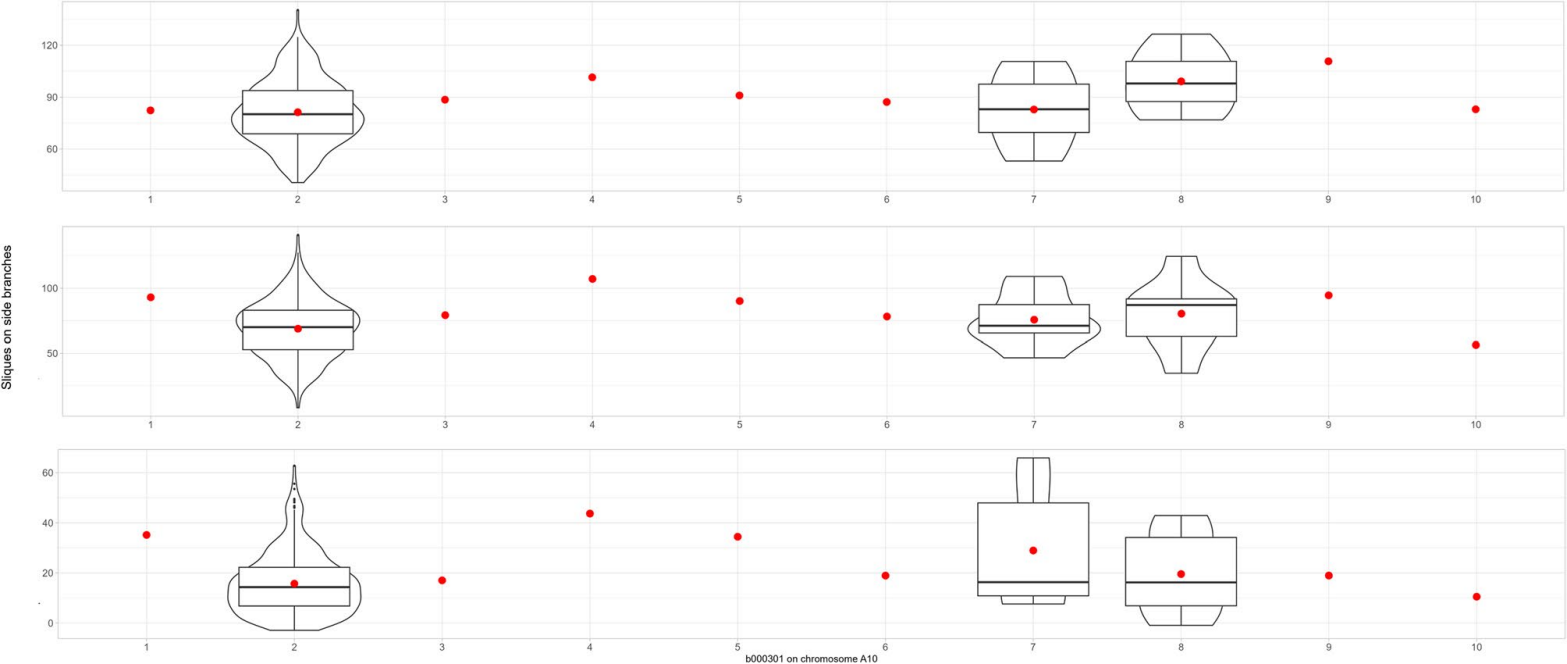
Effect size of haplotype b000301 on chromosome A10 on number of siliques on side branch (**A**) on level 1–5, (**B**) on level 6–10, (**C**) and 11–15. Results show ten different haplotype block alleles (1–10). Boxplots depict variance within the tested F1 Testhybids. Red dot indicated the median

**Fig. 5:**
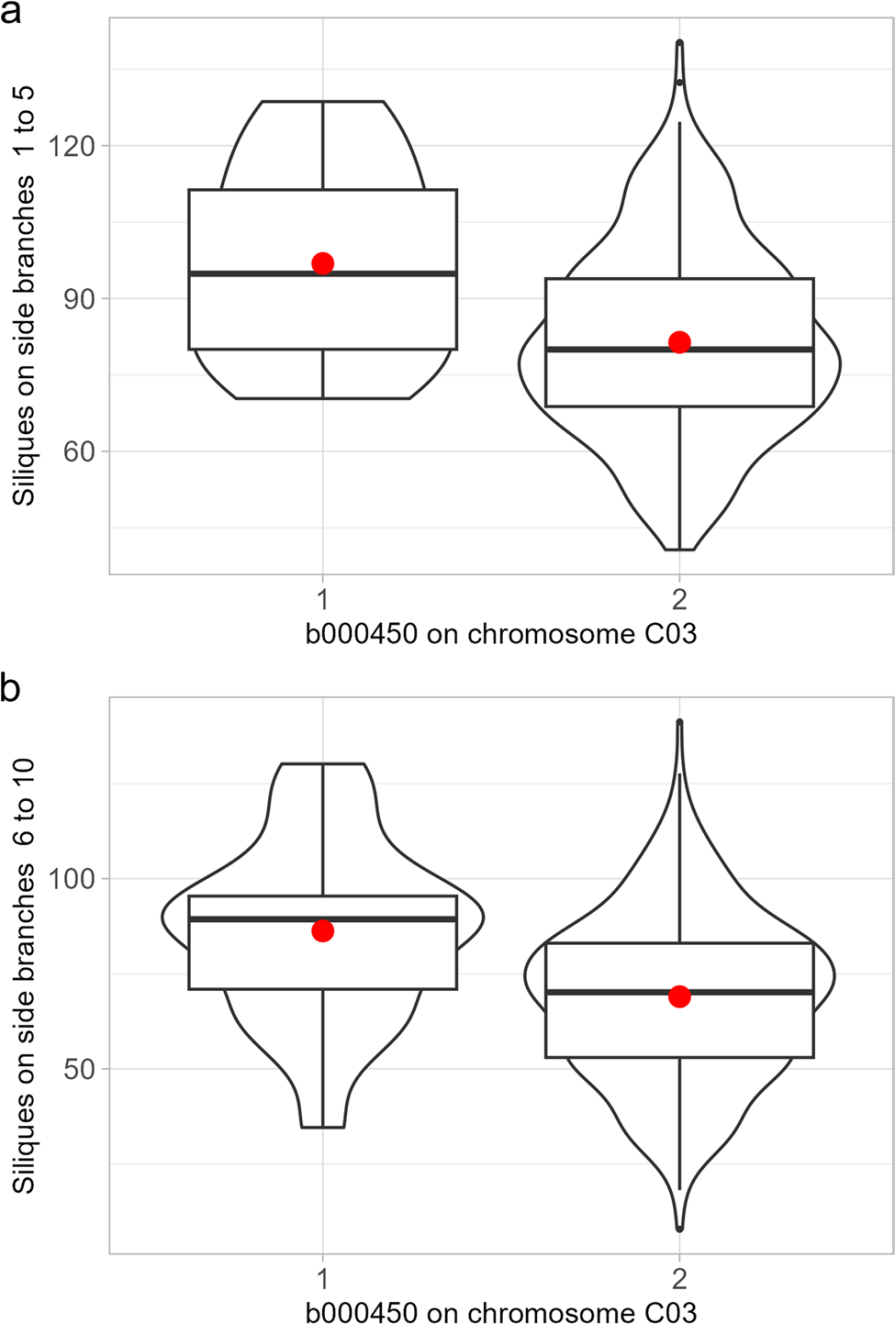
Effect size of haplotype b000450 on chromosome C03 on number of siliques on side branch (**A**) on level 1–5, (**B**) on level 6–10. Results show two different haplotype block alleles (1 vs. 2). Boxplots depict variance within the tested F1 Testhybids. Red dot indicated the median

Nevertheless, 10 blocks were found to be associated with multiple traits. Notably, haplotype block b000301 stood out for its influence on siliques across several side branch levels and the overall siliques per plant (Fig. 3, Tables S1 and S2). For example, this particular haplotype block, spanning a region of 5,007.14 kbp on chromosome A10, explained 1.88%, 1.29%, 7.54%, 3.04%, and 3.06% of the phenotypic variation for siliques on side branches 1 to 5, siliques on side branches 6 to 10, siliques on side branches 11 to 15, siliques on side branches 16 to 20, siliques per plant, and siliques per plant, respectively. The effect size of the haplotypes range between 56.36 and 107.24 siliques on side branch level 6–10 and 10.50 and 43.69 siliques on side branch level 11–15 (Fig. 4 and Supplementary Table S2). In addition to this haplotype block, a 5007.14 kbp block b000302 on chromosome A10 associated to silique numbers on various branch levels which for example explained more than 5% of the phenotypic variation for the number of siliques on side branches 11–15. Furthermore, a 2,452.06 kbp haplotype block (b000450) on chromosome C03 was also associated with silique numbers across various branch levels (Fig. 5).

####  Genome wide association studies for adjusted means for single years

To check whether some chromosome regions for the primary yield components have an environment-specific effect, i.e. a signal can only be detected for the trait in one of the two years, the GWAS was also carried out separately for the adjusted mean values from the respective years. The result shows that there is no haplotype block relationship that can exclusively be detected in one of the two years but not based on the values across both years. However, a significant relationship to the characteristic was found for 17 haplotype blocks, which was significant based on the data from the test year 2021 and the value across both years. Interestingly, with haplotype blocks b000555, b000315, b000549 the three blocks with the highest significance values 6.016, 4.564 and 4.545 respectively overlapped between the data based on both years (Supplementary Table S2). There are 7 significant haplotype blocks for the number of siliques on side branches 4 to 7. Only haplotype block b000555 appears as significant in three different GWAS, namely for the value for both individual years and for the value across both years. There was no significant relationship from a haplotype block to a trait based solely on the 2020 trial year data.

### Overlap with QTL from literature

Various blocks found to be significant here for silique number traits were also found to be of importance for architectural traits in literature (Table S2). For example, blocks b000039, b000200, b000246, b000450, b000455, b000549, b000641, b000674 and b000744 were also found to be associated to multiple branching related traits that were reported by Cai et al. 2016 and Zheng et al. 2017. Furthermore, the blocks b000075, b000168, b000210, b000211, b000247, b000450, b000677, b0000678 and b000744 coincide with QTL for branching angle reported previously [52, 54, 55].

## Discussion

Although winter oilseed rape has a high N acquisition capacity, a comparatively low nitrogen utilization efficiency leads to an overall limited total NUE. Unutilized nitrogen is not only a problem for ecosystems, as nitrate pollutes water bodies and nitrous oxide in the atmosphere drives climate change, but better utilization of nitrogen is also desirable from an economic and resource efficiency perspective. It allows to achieve a higher yield per united available nitrogen. Thereby, yield– as the relevant N sink– is not a single trait, but is determined by a variety of parameters that manifest in a changed plant architecture and different expression of primary yield components. To the best of our knowledge most study have not dissected seed yield in primary yield components nor have those traits be analyzed in two sets of experimental F1 hybrids. Here, we aimed to dissect the final plant architecture and to assess the genetic determinant of sink capacity traits under restricted N fertilizer application. Therefore, plant architectural traits as well as primary yield components of up to ten individual plants across two subsequent vegetation periods of 383 experimental F1 hybrids were assessed.

### Experimental conditions

NUE is not only dependent on the expression of the primary yield components, but these in turn are themselves sometimes previously determined by N availability as it is among the factors having a large effect on primary yield component expression. A systematic study encompassing GWAS, gene expression, microscopic and physiological analysis came to the conclusion that improving the biomass and nutrient accumulation is beneficial for enhanced silique number in rapeseed [58].In this regard, the present study assesses all traits on experimental F1 hybrids grown under restricted nitrogen fertilization.

Flowering marks the period where vegetative plant organs as leaves and stems gradually undergo a transition as N sink organ to N source. Therefore, developmental stages beginning with flowering are referred to as critical developmental stages, to distinguish high vs. low NutE genotypes [59]. From a plant physiology point of view an enhanced source-to-sink allocation of amino acids is an utmost relevant aspect and goes along an elevated number of branches and siliques under N-deficiency beginning from flowering/post-flowering stages [60, 61]. But the plant architectural traits are influenced at different development stages and some are already determined during vegetative developmental stages. With the beginning of the reproductive growth the shoot apical meristem transforms to the inflorescence meristem which produces floral primordia continuously [62]. It has to be considered that different parts of the plants are at different stages at the same day of the year. For example, the lower side branches are developing full siliques while on the upper part of the plant further side branches are still developing. This enables oilseed rape to have an outstanding compensatory capacity along the vegetation period. Given this complex indeterminate nature of oilseed rape in the present study in contrast to other studies we didn´t analyze plants that grew without interplant competition effects (e.g. in pot experiments in the greenhouse or from single/double rows with few individuals in the field), but the plants were taken from the core of a plot standardized in terms of plant density. Moreover, we conducted a distinct high-resolution dissection of number of side branches and number of siliques on each individual side branch along the vertical axis of the individual genotypes.

### Phenotypic assessment of traits contributing to nitrogen sink capacity

#### Angle and number of secondary branches

The angle of the side branches strongly depends on genes that encode a different influence by gravitropism [55] and are fixed very early during the vegetation. However, here we observed heritability to be negligible or zero (Table 1), and therefore did not analyze related traits in detail. The experimental F1 hybrids of the mother B developed more secondary side branches, in particular on side branch level 4 and 5. Here experimental F1 hybrids of mother A showed almost 22% and 28% less secondary branches.

#### Number of siliques

The fact that no substantial (even when significant) differences were found between the experimental F1 hybrids of both mothers for the total number of siliques per plant doesn´t proof that the trait is not relevant for yield determination per se as a different distribution of traits can be relevant. An earlier study indicated that with increasing environmental impact, simulated artificially by shading, the relevance of siliques on certain side branch levels can be shifted in accordance with the environmental impact [63]. Indeed, we have seen that the number of siliques increases from the lowest level to side branch 6, almost stagnates between side branch levels 6–10 and then decreases again (Table 1). This was seen for both sets of experimental F1 hybrids. Although the two experimental F1 hybrid sets differed only to a lower extent in the total number of siliques per plant, the distribution of the seeds along the vertical axis was different. The lower yielding experimental F1 hybrid set going back to mother A had approximately 16% fewer siliques on average across levels 1–10 but more siliques on side branch level 11 and higher. It can be argued that siliques on the upper level were not as productive and contributed only to a lower extent to seed yield. Thus, the side branches level harbors the most relevant sub-traits for yield determination as the number of siliques here showed the strongest differentiation between two experimental F1 hybrid sets, the strongest correlation to seed yield and highest heritability. The tendency of the higher-yielding set to form more siliques at the middle level is similar to the picture of the tendency towards secondary branch development. It can be speculated that mother B inherits a predisposition that leads to a stronger proliferation of corresponding tissues at the relevant developmental stages. Further, the GWAS for the number of siliques on side branches based on the single year 2021 also shows a higher density of significant haplotype blocks on side branches levels 4–7. Here we found seven significant haplotype blocks, while for side branches 8 to 14 six haplotype blocks were found and for side branches below side branche level 4 only one haplotype block.

#### Seeds per silique

For the number of seeds per silique, their potential is initially determined by ovule initiation, but then their realization is restricted. Despite some loci regulating silique length [64] and ovule abortion was fine mapped [65], the exact regulation of both the potential setting and abortion of the individual tissues or organs is not fully understood. The influence of nutrient supply, fertilization and phytohormone modulation is considered as certain [66, 67]. For example, the number of ovules, seeds and siliques is dependent on photosynthesis [68] and on the influence of environmental factors such as various stresses. 

#### TSW

TSW is the last trait that is altered along the vegetation period and consequently, can compensate for shortcoming in expression of earlier traits. For example, 70% shading imposed during flowering, as a measure to simulate an altered source-sink relationship– let to approx. doubling of TSW fully compensating yield penalty of the temporal shading [27]. Given the small variation in TSW we conclude that during this experiment F1 hybrids showed no substantial difference in yield compensation via different TSW expression and thus most differences in yield is rather attributed to variation in traits discussed above.

#### Limitation of this study

The interaction of aforementioned traits is under polygenic control and therefore poses a great challenge for plant breeding. According to the theory, it is assumed that the individual components have a higher heritability as sub-traits and that their determinants can therefore be more easily understood and processed in breeding when considered individually [53]. The original hypothesis that sub-traits per se have a higher heritability than the yield per se cannot be proven here. Seed yield showed the highest heritability of all traits recorded (h^2^ = 0.41). However, a direct comparison is not possible. While yield and TSW were measured by threshing the whole plot, all other traits were determined on single plants from the plot and are subject to a large statistical residual, which has a heritability reducing effect. Measuring the yield on individual plants was not methodologically possible, as we removed the plants shortly before maturity to prevent the siliques from bursting and to record all parameters very accurately. We are aware that the sampling of 2 × 5 plants in two consecutive years at one location is limited in terms of test environments. The sampling size of 10 plants is small. However, the fact that we achieved a heritability for the traits with the strongest correlation to yield (e.g. number of siliques at the medium level) that was on par with the TSW measured from the plot threshing without sampling error underscores that the results are still highly relatable. In addition, the results are confirmed by overlap with some known QTLs, which is further evidence of the reliability of the data.

Furthermore, we would like to remind that due to the labor-intensive nature of the detailed dissection of plant architectural traits, this study is based on data from only one location in two years, i.e. two environments. Thus, the study is limited in geographic and environmental scope and could not capture expected complex genotype by environment interactions and focuses on per se effects.

### Genetic dissection of plant architectural traits and primary yield components

For yield related traits a considerable number of QTL were identified previously [69]. But even for the sub-traits investigated in this study a vast number of QTLs were published elsewhere [69]. More than 90 loci have been published for the silique number [54, 58, 62, 70] and except one on all *B. napus* chromosomes QTL coding for plant architectural trait were published, underlining the genetic complexity of yield formation in oilseed rape [58]. Therefore, map-based cloning approached remain from limited use, not at least due to the moderate effects shown by the individual loci. It is proposed that construction of haplotypes improves the identification of genetic determinants of quantitative traits as yield and yield stability [71, 72]. In the present study we extend our knowledge of the genetic control of yield and its components under N constraints, by analyzing the effect of haplotypes on N sink/yield related traits followed an identification of responsible. Although the transfer to other populations and the use in commercial breeding programs should be critically discussed and the regions need to be further investigated, given the complexity of the traits, up to 9% explained phenotypic variation by single haplotype blocks seems to be considerable. In particular, since the number of siliques was counted separately at all levels of the lateral branches, the identification of significant associated genomic regions per GWAS can be considered an independent analysis. The fact that individual haplotype blocks as b000301 show a significant signal at all levels and across but also only within 2021 years data underlines the general relevance of these genomic regions for the formation of siliques at different levels of the canopy package.

### Comparison of identified loci with other findings

Earlier studies have already dealt with the plant architecture and the primary yield components of oilseed rape and carried out genetic mapping. Overlap with studies that conducted experiments independently of this study can be seen as additional confirmation of significant identified regions.

#### Haploblocks associated to multiple traits within this study

The largest number of overlapping loci from our study with the literature was found for the study of Cai et al., (2016) [53]. A comparison of the chromosomal regions detected here with significant signals for this study revealed that two haplotype blocks are located in a region that was also assigned significance by Cai et al. (2016) [53]. This concerns b000450 and b000678, both of which are relevant for the number of siliques on side branches 1–5 and are also associated with a variety of architectural traits by Cai et al. 2016 [53] (Table S2).

Although the phenotypic variation explained by each QTL for the individual trait is small, it is worth noting that some haplotype blocks show significant associations with multiple traits, for example b000301 on chromosome A10 for the number of siliques on side branches 1–5 and 11–15, respectively. Considering that the traits were recorded separately, and the QTL were calculated independently, the multiple occurrences of the same block for the same trait at different side branch levels strengthen the evidence that those loci are to be considered for the trait expression.

#### Overlap of haplotype blocks with loci in other studies for branch angle and number of siliques on side branches

Plants that have a narrow angle of lateral shoots form a rather steep and compact canopy architecture. This also has an indirect influence on the formation of other yield parameters, due to altered transmission of light rays and reduced spatial competition. A study investigating the genetic architecture of side branch angle [55] has identified in 520 diverse genotypes 56 loci significantly associated with the angle. Interestingly, we found haplotype blocks for number of siliques (b000210 and b000211 on Chromosome A07, b000247 on A08, b000450 on C03, b000678 on C08 and b000744 on C09) in the same region as previously described QTLs for branch angle [55]. Further, b000211 showed also to be significant for siliques on side branche level 12 in case only data from year 2021 were used for the GWAS. Haplotype block b000744 on chromosome C09 was one out of two haploblocks for number of siliques on side branches that was also previously identified in another GWAS based on 472 diverse accessions [52]. Further overlaps with previously identified QTL [54] were also identified for haplotype block b000168 on chromosome A05. Given the overlaps across multiple studies and unrelated populations, these genomic regions are likely to be involved in controlling plant architecture and silique formation. Nonetheless, because a relatively liberal threshold was used to detect marker–trait associations, it is likely that these traits are largely quantitatively. Furthermore, it can only be speculated whether the observation that many of the common QTLs for the number of siliques on the majority of upper side branches (side branches 9, 10, 11, 12, 14, 16, only two overlaps for side < 9) are related to the fact that the steeper angles, the later developed side branches are placed in a spatially favorable position to form a high number of pods.

#### Outlook

This study provided a detailed insight into the yield-determining factors of winter oilseed rape and revealed genetic variation and haplotype blocks with significant influence on yield-relevant architectural traits. In future, candidate genes should be narrowed down in such regions responsible for expression of relevant traits to facilitate targeted selection in breeding programs. In particular, haplotype blocks that show a signal for several traits in this study and/or those that also overlap with the findings of other studies become relevant for applied breeding. For example, they can be used to develop diagnostic markers that enable breeding programs– in addition to genome-wide prediction models– to select genotypes carrying the positive alleles in early generations.

The study also revealed differential effects of maternal and paternal F1 hybrid parents. In a more balanced factorial, future studies could investigate the influence of heterosis and general combining ability of the parents.

## Supplementary Information

Supplementary Material 1.

Supplementary Material 2.

Supplementary Material 3.

Supplementary Material 4.

## Data Availability

Genome wide marker data (SNP) used within this study are available in Supplementary Table S1. Phenotypic data will be made available upon request.

## Abbreviations

BnNAM: B. napus nested association mapping population
LD: Linkage disequilibrium
GWAS: Genome Wide Association Study
N: Nitrogen
NupE: Nitrogen uptake Efficiency
NutE: Nitrogen Utilisation Efficiency
NUE: Nitrogen Use Efficiency
OSR: Oilseed rape
QTL: Quantitative Trait Loci
SNP: Single Nucleotide Polymorphism marker
TSW: Thousand Seed Weight
RILs: Recombinant inbreed lines
