# Supplementary figures and images for: Genetic dissection of plant architecture reveals haplotypes controlling sink-related traits in oilseed rape under limited nitrogen fertilization
Source: BMC Plant Biol. 2025 Aug 27;25:1138. doi: 10.1186/s12870-025-07035-2 (PMC12382299; doi:10.1186/s12870-025-07035-2)

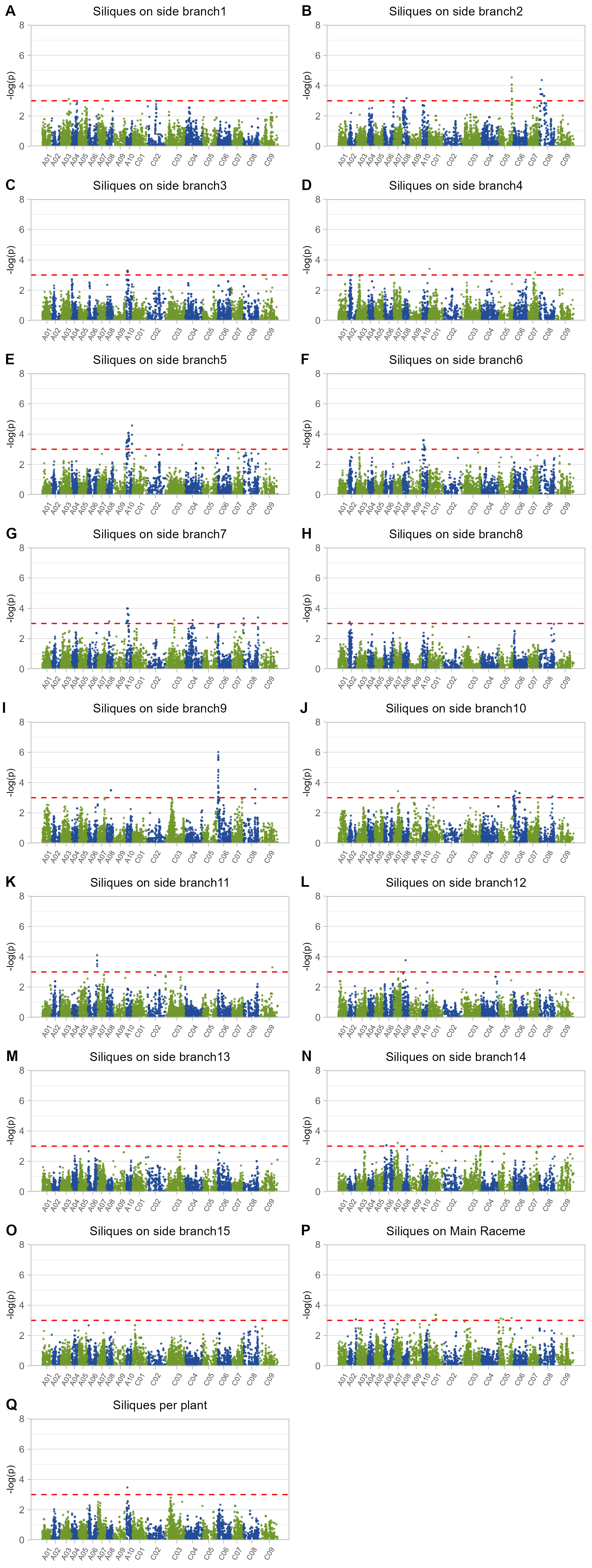

Supplement: Supplementary file 1 — Supplementary Material 1. [file 12870_2025_7035_MOESM1_ESM.png]

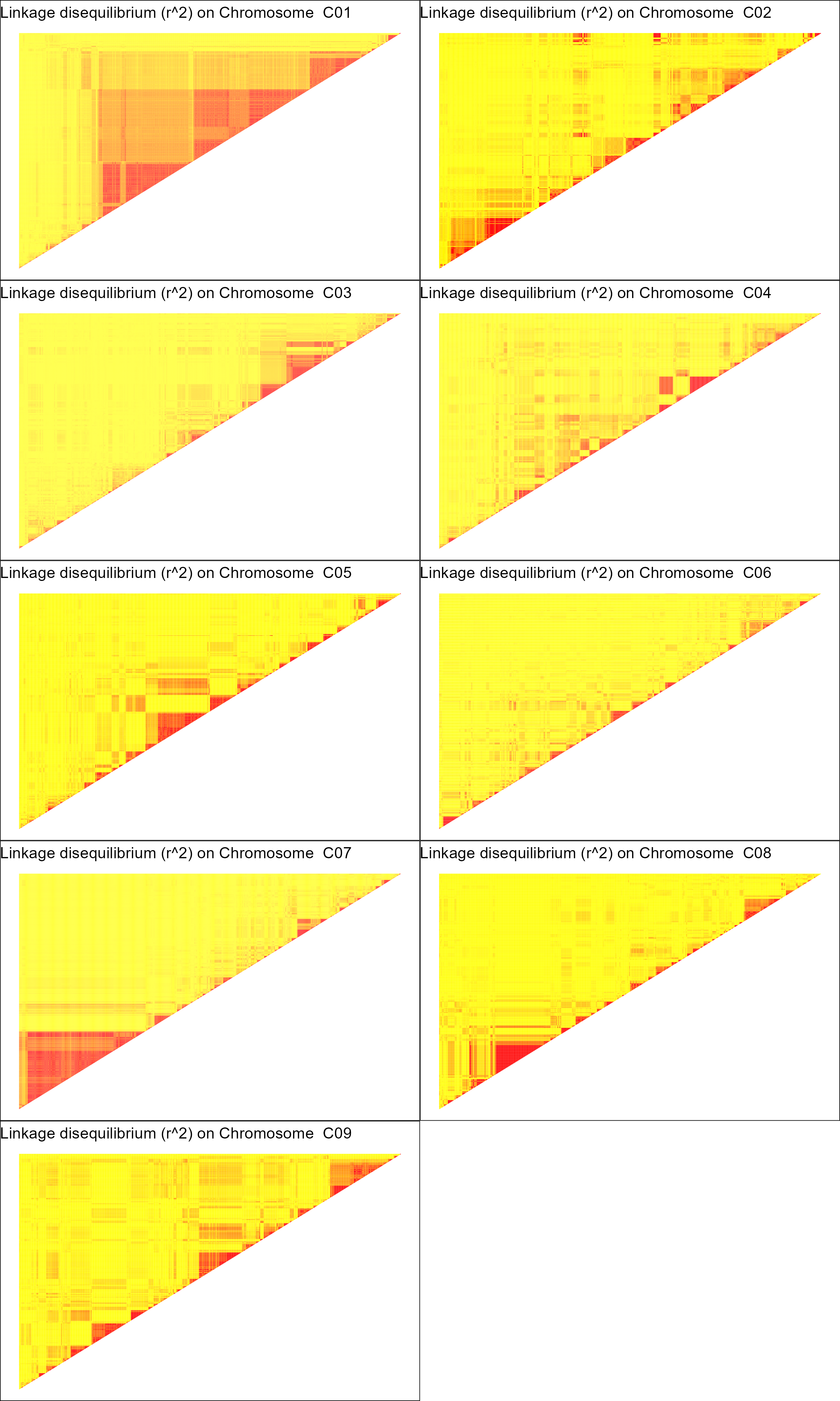

Supplement: Supplementary file 2 — Supplementary Material 2. [file 12870_2025_7035_MOESM2_ESM.png]

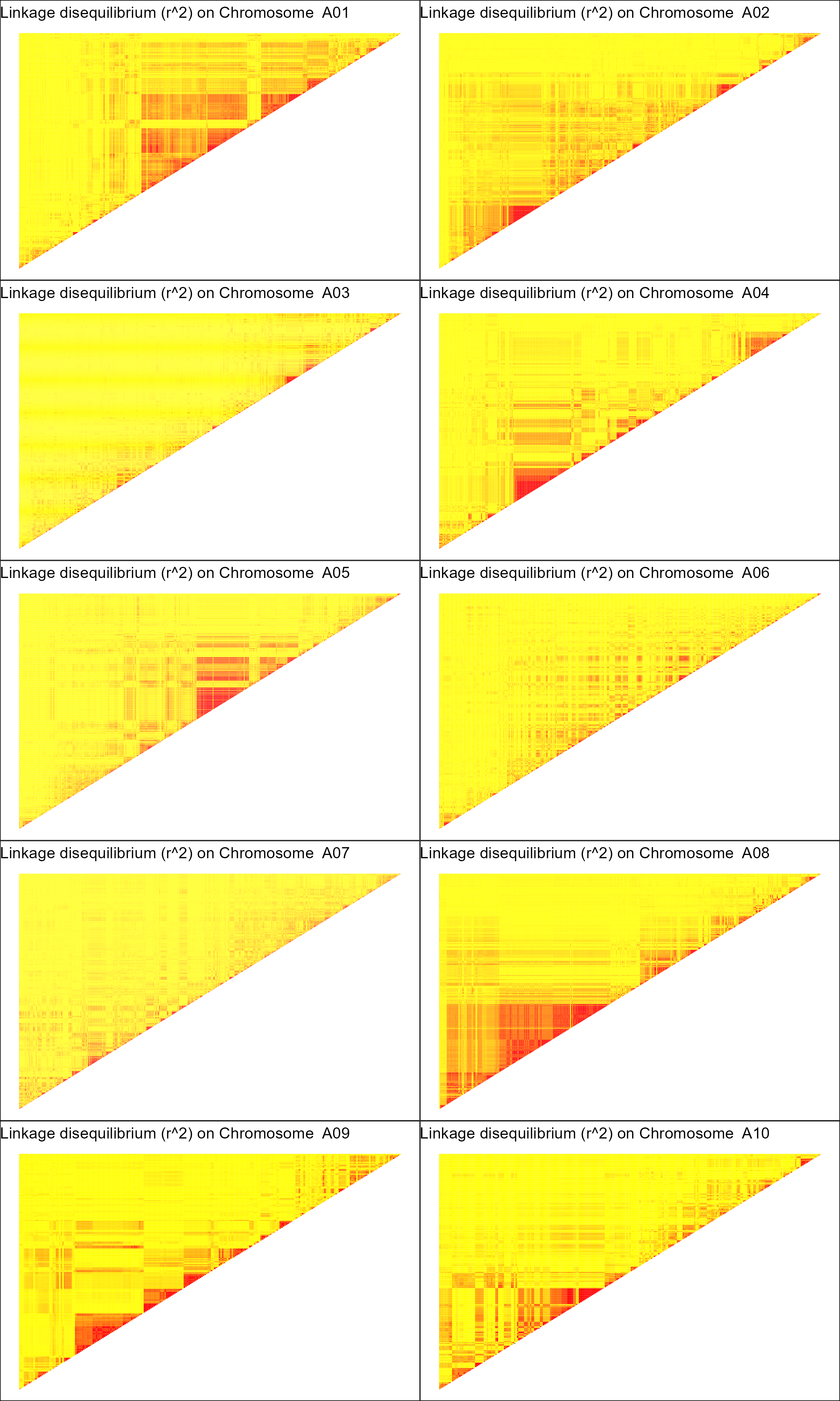

Supplement: Supplementary file 3 — Supplementary Material 3. [file 12870_2025_7035_MOESM3_ESM.png]
